# Possible Anti-Pain Vaccines: A Narrative Review of Emerging Strategies and Clinical Prospects

**DOI:** 10.3390/vaccines13090909

**Published:** 2025-08-27

**Authors:** Yuri Chaves Martins, Luciana Pereira De-Sousa, Peyton J. Murin, Hamed Sadeghipour, Cláudio Tadeu Daniel-Ribeiro

**Affiliations:** 1 Department of Anesthesiology, Saint Louis University School of Medicine, St. Louis, MO 63110, USA; hamed.sadeghipour@health.slu.edu; 2 *Laboratório de Pesquisa em Malária, Instituto Oswaldo Cruz, Fundação Oswaldo Cruz (Fiocruz) and Centro de Pesquisa, Diagnóstico e Treinamento em Malária*, Fiocruz, and *Secretaria de Vigilância em Saúde e Ambiente* (SVSA), Ministry of Health, Rio de Janeiro 21040-900, RJ, Brazilmalaria@fiocruz.br (C.T.D.-R.); 3 Department of Neurology, Saint Louis University School of Medicine, St. Louis, MO 63104, USA; peyton.murin@slucare.ssmhealth.com

**Keywords:** analgesia, calcitonin gene-related peptide, chronic pain, immunotherapy, neuroimmunology, nerve growth factor, anti-pain vaccines, transient receptor potential vanilloid 1

## Abstract

Chronic pain affects millions of individuals globally and continues to pose a major burden on patients and healthcare systems. Traditional analgesics, such as opioids and nonsteroidal anti-inflammatory drugs, often provide only partial relief and are frequently associated with significant side effects and risks of misuse. In recent years, vaccines that target molecules involved in pain signaling have emerged as an innovative therapeutic strategy. These vaccines aim to induce long-lasting immune responses against key mediators of nociception, including nerve growth factor (NGF), calcitonin gene-related peptide (CGRP), substance P, and voltage-gated sodium channels such as Nav1.7. By promoting the production of specific antibodies, anti-pain vaccines have the potential to achieve analgesic effects with longer duration, reduced need for frequent administration, and improved accessibility. Multiple vaccine platforms are under investigation, including virus-like particles, peptide-protein conjugates, and nucleic acid technologies. Although preclinical studies have shown promising efficacy and safety profiles, clinical evidence is still limited to early-stage trials, particularly for migraine. This narrative review summarizes current knowledge on therapeutic vaccines for pain, discusses the immunological and technological advances in the field, and outlines future directions.

## 1. Introduction

According to the International Association for the Study of Pain (IASP), chronic pain is “pain that lasts or recurs for longer than three months and is associated with significant emotional distress and/or functional disability” [1]. Chronic pain is one of the most prevalent medical conditions, affecting approximately 30% of the world population [2] and impacting 50.2 million adults in the United States alone [3]. Chronic pain has a detrimental effect on quality of life for patients and poses a significant cost to the medical system [3]. Traditional analgesics (e.g., NSAIDs, anticonvulsants, or opioids) often provide incomplete relief or cause significant side effects, including potential misuse. As such, there is an urgent need for improved therapeutics.

Chronic pain encompasses a diverse set of pathophysiological mechanisms that influence both the selection and expected efficacy of therapeutic targets. At the peripheral level, nociceptor sensitization occurs when primary sensory neurons in the dorsal root or trigeminal ganglia develop reduced activation thresholds and heightened responsiveness to stimuli due to injury, inflammation, or disease [4]. This process is often driven by the upregulation of ion channels, receptors, and inflammatory mediators—such as NGF, TRPV1, and certain cytokines—that enhance afferent signaling [5,6]. These changes may be reversible in acute settings but can become maladaptive, establishing a foundation for chronic pain states.

Central sensitization refers to increased excitability of neurons within the spinal dorsal horn and supraspinal centers, resulting in amplification of pain signals and expansion of receptive fields [7]. This phenomenon is maintained by sustained peripheral input, altered inhibitory control, and activity-dependent synaptic plasticity, and it can persist even after the resolution of the original peripheral insult [8]. Central sensitization is a hallmark of many neuropathic and nociplastic pain conditions, and molecular drivers such as CGRP, SP, and specific glutamatergic pathways contribute to this hyperexcitability [9,10].

Neuro-immune interactions further shape the chronic pain landscape. Inflammatory pain is characterized by the release of pro-inflammatory cytokines (e.g., TNF-α, IL-6) from immune and glial cells, which modulate nociceptive transmission at both peripheral and central levels [11]. Neuropathic pain arises from direct injury to the nervous system, involving maladaptive immune responses and persistent activation of microglia and astrocytes [12]. Nociplastic pain, exemplified by conditions such as fibromyalgia, is defined by altered nociception without clear evidence of tissue or nerve damage, often associated with dysregulated descending modulation [13]. Understanding whether a given antigen primarily affects peripheral sensitization, central sensitization, or immune-mediated processes can provide insight about which patient subgroups might benefit from vaccination strategies. For example, NGF blockade is most likely to attenuate inflammatory and peripheral sensitization mechanisms, whereas CGRP-targeted approaches may have greater relevance in migraine and other centrally mediated pain syndromes. However, it is important to acknowledge that many patients experience mixed mechanisms at the same time.

Biological therapies like monoclonal antibodies (mAbs) against nerve growth factor (NGF) (e.g., tanezumab) and calcitonin gene-related peptide (CGRP) (e.g., erenumab) have shown promise in the treatment of chronic pain conditions such as osteoarthritis and migraines [14,15]. NGF is upregulated in osteoarthritis, enhancing signaling via Tropomyosin receptor kinase A (TrkA) and promoting central sensitization [16]. Neutralizing NGF with mAbs can significantly reduce pain in some osteoarthritis and low-back pain patients [17,18]. Similarly, blocking CGRP, a neuropeptide crucial to migraine pathophysiology, prevents migraine attacks in many sufferers [19]. These successes underscore the potential for harnessing the immune system to target pain modulators. However, biological therapies are costly and require repeated administration, limiting accessibility. These limitations have spurred interest in therapeutic vaccines for pain management, defined as active immunizations designed to induce the body’s own immune system to neutralize pain-related molecules or drugs on a long-term basis.

**Figure 1:**
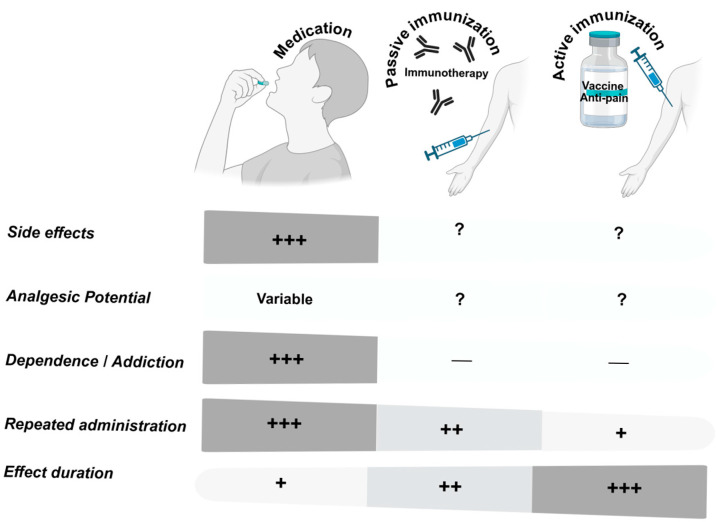
Comparative overview of therapeutic strategies for chronic pain. Different strategies for managing chronic pain include medication-based treatments, passive immunization (such as monoclonal antibody therapies), and active immunization (such as anti-pain vaccines). These approaches differ across several clinical parameters. Medications are typically associated with a higher incidence of side effects, a greater need for repeated dosing, increased risk of dependence or addiction, and shorter duration of effect. In comparison, passive and active immunization strategies may provide longer-lasting relief, reduced dosing frequency, and a lower risk of dependence. However, the side effect profiles and analgesic potential of immunotherapies are still not fully established. (+) minor; (++) moderate; (+++) major; (—) absent; (?) unknown.

Anti-pain vaccines leverage the principle that generating endogenous antibodies against key mediators can modulate nociceptive signaling. The rationale builds on two key observations: (1) specific molecular targets play pivotal roles in pain pathways, and (2) passive immunization (monoclonal antibodies) against these targets can produce analgesia. By inducing a patient’s immune system to produce antibodies, it is theoretically possible to mimic the effect of therapeutic antibodies but with a longer duration. Once a robust antibody titer is achieved, protection/analgesia may persist for months to years with only periodic boosters, improving adherence. Therefore, such vaccines could potentially offer sustained pain relief at a lower cost than chronically delivered drugs [17,20]. Furthermore, vaccines are scalable and cost-effective, potentially allowing for improved accessibility and reduced treatment costs [10]. Nevertheless, the application of vaccination to the treatment of pain is not without challenges. Advances in vaccine design (e.g., conjugating self-antigens to immunogenic carriers or virus-like particles) have made it feasible to generate high-titer antibodies [21], but many pain targets are self-proteins requiring breaking immune tolerance. (Figure 1)

In summary, anti-pain vaccines offer potential for long-lasting and effective pain relief. However, there are challenges that must be overcome. Here, we review the preclinical and clinical evidence for therapeutic vaccination in pain management. In doing so, we provide a summary of the status of the field and areas for future research.

## 2. Anti-Pain Vaccine Targets

Therapeutic anti-pain vaccines under investigation focus on a range of molecular targets known to mediate nociception, analgesia, and pain sensitization. Evidence supporting the viability of these vaccines comes primarily from preclinical studies in animal models, with a few early-stage clinical investigations underway. Below we summarize the most studied targets, their scientific rationale, and the seminal and most recent clinical studies.

### 2.1. Nerve Growth Factor (NGF)

NGF is a neurotrophin essential for nociceptor sensitization and survival of certain sensory neurons. It binds TrkA receptors on pain fibers, leading to heightened pain signaling and inflammatory hyperalgesia [16]. Elevated NGF levels are observed in chronic pain states (e.g., osteoarthritis, intervertebral disk degeneration), and in humans, an anti-NGF antibody (tanezumab) produced significant analgesia in clinical trials of osteoarthritis and chronic back pain [22]. These trials also revealed a dose-dependent side effect of accelerated joint degeneration in a subset of patients, highlighting NGF’s role in joint homeostasis [17].

A vaccine strategy against NGF has been pioneered in animal models. A landmark study by von Loga et al. provided the first direct evidence that active immunization can attenuate chronic pain [23]. By coupling the NGF protein to an immunogenic virus-like particle (VLP) carrier, von Loga et al. were able to successfully break self-tolerance in a murine model [23]. They then applied this vaccination in a murine model of osteoarthritis, resulting in high anti-NGF antibody titers and reversibly alleviated pain behaviors (improved weight-bearing on the affected limb), both in prophylactic and therapeutic vaccination regimens [23]. Pain relief correlated with peak antibody levels and was significant compared to controls; however, regular booster shots were required to maintain antibody titers and pain relief [23]. Histological analysis confirmed that the vaccine did not worsen joint pathology relative to controls [23]. Functionally, serum from vaccinated mice could neutralize NGF activity in vitro, indicating that the antibodies were capable of blocking NGF-TrkA signaling [23]. These findings established proof-of-concept that active immunization against NGF can safely recapitulate the analgesic effect of NGF-neutralizing antibodies in chronic pain. No overt toxicity or motor deficits were noted in the animals, supporting the safety at least in the short term. However, further study is needed to assess long-term safety and efficacy of NGF-targeted vaccination in human patients [24]. These findings have spurred interest in translating an NGF vaccine toward human trials, especially for osteoarthritis pain, where anti-NGF biologics showed efficacy but were limited by cost and safety issues. It should be noted that all evidence to date remains preclinical, and transitioning an NGF vaccine to the clinical setting would require overcoming significant safety concerns [23].

### 2.2. Substance P (SP)

SP is an 11-amino-acid neuropeptide of the tachykinin family that transmits pain signals and promotes neurogenic inflammation. It binds neurokinin-1 (NK1) receptors on neurons, immune cells, and vasculature, causing plasma extravasation and pain sensitization. SP is widely distributed in the dorsal horn and peripheral nerves and has been implicated in the pathogenesis of various chronic pain conditions (e.g., inflammatory arthritis and neuropathic pain) [25]. This made the SP/NK1 pathway a promising analgesic target in the 1990s–2000s. Indeed, in animal models, NK1-receptor antagonists reliably reduced pain behaviors, especially in inflammation-induced hyperalgesia, supporting SP’s role in nociceptive facilitation [26]. However, except for acute post-surgical pain [27], clinical trials of NK1 antagonists failed to show significant chronic pain relief in humans [28,29,30,31]. Based on these findings, the concept of a ‘species gap’ was proposed, suggesting that although blockade of substance P (SP) signaling effectively attenuates pain responses in rodent models, it does not provide adequate analgesia in chronic pain conditions [28]. Nevertheless, the underlying biology remains robust, as SP release contributes to pain and central sensitization in conditions such as fibromyalgia and complex regional pain syndrome, with elevated SP levels correlating with greater pain intensity [25]. Therefore, a vaccine against SP could, in theory, induce neutralizing antibodies to sequester SP in the periphery and block its pro-pain actions. While no peer-reviewed studies of an SP vaccine in animals have been reported to date, a search of the patent literature reveals a promising bivalent vaccine targeting both NGF and SP that has been tested in dogs with naturally occurring osteoarthritis [32]. This recombinant fusion protein, composed of canine NGF and SP sequences, induced strong antibody responses and led to substantial reductions in pain scores, along with significant improvements in mobility and daily function. The vaccine was well tolerated in dogs and showed no major adverse effects in supporting pre-clinical safety studies in mice and horses. By concurrently neutralizing two key nociceptive mediators, this dual-target strategy offers a novel and potentially disease-modifying approach to long-term pain control. Another challenge to produce a SP vaccine to use in humans will be to generate antibodies that reduce pathological SP signaling without disrupting its normal modulatory functions, as SP also has anti-nociceptive effects under certain conditions [33]. In summary, Substance P remains an intriguing but as-yet unproven vaccine target. Future work may explore SP conjugate vaccines or NK1 receptor vaccines, possibly in specific pain syndromes with clear SP involvement.

### 2.3. Calcitonin Gene-Related Peptide (CGRP)

CGRP is a 37–amino acid neuropeptide that plays a central role in migraine and other pain disorders. It is released from trigeminal sensory fibers during migraine attacks, producing potent vasodilation and neuroinflammation in the meninges and contributing to headache pain [34]. It also modulates pain transmission in the spinal cord and periphery [35]. The calcitonin gene-related peptide (CGRP) pathway is among the most well-validated targets in pain therapeutics, as both small-molecule CGRP receptor antagonists (commonly known as “gepants” such as ubrogepant and atogepant) and monoclonal antibodies targeting CGRP or its receptor have shown significant efficacy in migraine prevention [36]. For example, Phase II/III trials showed that anti-CGRP antibodies (e.g., galcanezumab, fremanezumab) or receptor-blocking antibodies (erenumab) reduce monthly migraine days by ≥50% in a large fraction of patients, with good safety profiles [19,37]. These successes make CGRP an attractive target for a vaccine approach in migraine prophylaxis. A CGRP vaccine would aim to induce durable anti-CGRP antibodies that neutralize the peptide when released during headache onset. Per a company press release, such a vaccine, UB-313, is reported to have been developed by Vaxxinity [38]; however, no peer-reviewed clinical data are available at this time. In preclinical studies, UB-313 immunizations generated high titers of anti-CGRP antibodies in rodents and non-human primates, which bound human CGRP with high affinity and blocked CGRP-induced signaling [39]. Vaccinated rats showed reduced pain behavior in a capsaicin-induced trigeminal pain model, supporting the vaccine’s mechanism [39]. In addition, rats immunized with UB-313 developed high titers of antibodies that bound CGRP that blocked its interaction with the CGRP receptor in vitro and had attenuated hypersensitivity and reduced meningeal vasodilation compared to controls, indicating functional neutralization of CGRP [39]. Non-human primates (cynomolgus monkeys) were also tested for safety and immunogenicity with UB-313, which induced antibodies in monkeys without significant adverse effects reported, and the antibodies cross-reacted with human CGRP, supporting translational potential. A phase I trial is underway (NCT05477095), which will assess whether these antibodies can sequester enough CGRP to reduce migraine frequency. If successful, this would represent the first clinical proof-of-concept for an anti-pain vaccine. Beyond migraine, a CGRP vaccine could potentially be tested for other CGRP-mediated pain syndromes (such as cluster headache or postoperative headache), although migraine remains the primary indication.

Although short-term studies with anti-CGRP mAbs have not revealed major safety issues, since CGRP also has important physiological roles (e.g., vasodilatory protection of cardiac and cerebral vessels), the long-term safety of chronically neutralizing CGRP via vaccination will require close monitoring [40]. If successful, a CGRP vaccine could provide a cost-effective, infrequently dosed alternative to monthly antibody injections for migraine sufferers.

### 2.4. Transient Receptor Potential Vanilloid-1 (TRPV1)

TRPV1 is a non-selective cation channel expressed by nociceptive neurons, best known as the receptor for capsaicin (the heat in chili peppers). It is a polymodal detector of noxious stimuli—activated by heat > 43 °C, acidity, and certain lipid mediators—and is a critical mediator of peripheral pain sensation [41]. TRPV1 contributes to inflammatory pain hypersensitivity (e.g., in arthritis or neuropathy) by becoming sensitized and overactive in injured tissues [42]. Genetic knockout of TRPV1 in mice results in loss of heat pain and reduced inflammatory hyperalgesia, confirming its key role in nociception [43]. In parallel, agonist desensitization of TRPV1-expressing fibers with high-dose capsaicin has been successfully used for pain relief (e.g., topical 8% capsaicin patches for neuropathic pain) [44]. These characteristics established TRPV1 as a prominent target for analgesic development, leading to the creation of numerous TRPV1 antagonists [44]. A recent example includes the antagonist (AMG9810), which, when injected into the spinal cord of mice, reversed mechanical allodynia and thermal hypersensitivity [45]. While TRPV1 antagonists blocked pain in animal models, most clinical trials faltered due to development of hyperthermia (fever) caused by systemic TRPV1 inhibition [44,46,47]. Strategies to overcome this side effect such as the use of peripherally restricted antagonists or modality-specific blockers are under investigation [41,48]. A vaccine targeting TRPV1 could theoretically work by generating antibodies that reduce channel activity. However, TRPV1 is an integral membrane protein with multiple epitopes, making it a complex immunogen [49]. Any effective antibody would likely need to bind an extracellular domain and either block the channel’s gating or mark the expressing neuron for immune down-modulation. To date, no TRPV1-targeted vaccine has been reported, but related approaches hint at feasibility. For instance, researchers have raised nanobodies and monoclonal antibodies against TRPV1’s extracellular regions that modulate its function [50]. Another possibility is an autoimmune approach whereby controlled induction of a cytotoxic T-cell response against TRPV1-expressing neurons could mimic the effect of capsaicin ablation. Such an approach would carry significant risks and challenges in specificity. Another interesting proxy is the use of repeated low-dose capsaicin injections in animals to induce endogenous anti-capsaicin antibodies. One study noted that guinea pigs receiving multiple capsaicin-BSA immunizations produced antibodies that reacted with TRPV1-expressing neurons and slightly increased their heat pain threshold (indicative of partial TRPV1 blockade) [51], but this approach is still very experimental. Future vaccine designs might leverage engineered fragments of TRPV1 as immunogens to elicit blocking antibodies, potentially providing analgesia without the hyperthermic side effect seen with small-molecule antagonists. In addition, any TRPV1 vaccine strategy must be carefully tuned to avoid causing irreversible damage to sensory neurons or loss of normal temperature sensation.

### 2.5. Voltage-Gated Sodium Channel Nav1.7 (Nav1.7)

Voltage-Gated sodium channels (Nav) are involved in the generation and propagation of electrochemical impulses along nociceptors [52]. Nine Nav subtypes have been described, with Nav1.7, Nav1.8, and Nav1.9 being almost exclusively found in peripheral neurons. Nav1.7 is encoded by SCN9A. It is often called the “pain channel” because human genetic studies have shown it to be absolutely crucial for pain signaling, as loss-of-function mutations in SCN9A cause congenital insensitivity to pain, whereas gain-of-function mutations cause extreme pain disorders [53]. Nav1.7 is localized at nociceptor nerve endings and axons, where it amplifies depolarizations to initiate action potentials. Accordingly, Nav1.7 emerged as a key target for analgesic drug development, with the ultimate goal of creating a therapy that replicates congenital insensitivity to pain without causing additional side effects. Despite intense efforts over 15+ years, no selective small-molecule Nav1.7 blocker has yet succeeded clinically [54]. Many compounds showed good in vitro block but minimal analgesia in humans, potentially due to compensatory mechanisms or insufficient in vivo blockade. An immunotherapeutic approach to Nav1.7 could involve raising antibodies that bind the channel and inhibit its function. This concept is supported by precedent, as biological toxins such as tetrodotoxin (TTX) and certain peptide toxins are known to bind Nav channels with high affinity and specificity [55]. Researchers have also developed monoclonal antibodies and even single-domain antibodies (nanobodies) that target Nav1.7’s voltage-sensor domains and can allosterically inhibit channel opening [50,56]. One such monoclonal antibody was reported to effectively reduce pain in rodent models by selective Nav1.7 blockade, though translating this to humans is complicated by the need for the antibody to access neuronal membranes and by potential off-target binding to other Nav channels [57]. A vaccine against Nav1.7 would likely entail immunizing with a portion of the channel protein (for example, an extracellular loop peptide or an engineered domain). The goal would be to elicit antibodies that either block the channel’s ion conductance or mark Nav1.7-expressing neurons for immune attack. Achieving specificity is challenging given the homology of Nav1.7 with other sodium channels. Moreover, a robust immune response might risk autoimmune neuropathy. To mitigate this, future vaccine designs could focus on unique epitopes from Nav1.7 (perhaps discovered through phage display or computational epitope mapping) that are not present in Nav1.8 or other subtypes. While still hypothetical, a Nav1.7 vaccine represents a bold approach to essentially “immunize against pain” by knocking down one of the body’s fundamental pain signaling proteins. Recent advances in isolating inhibitory nanobodies against Nav1.7 [53] suggest that the immune system may produce anti-Nav1.7 antibodies if given a suitable antigen. Those nanobodies also significantly reduced pain responses in mouse models of inflammatory pain. Any progress here would be paradigm-shifting, but safety will be the paramount concern.

### 2.6. Adjunct and Co-Therapy Strategies

The opioid and cannabinoid systems are not pain mediators per se, but rather modulatory systems that can be targeted via vaccines for indirect pain management benefits. One strategy focuses on vaccines against opioid drugs (such as heroin, fentanyl, oxycodone) to prevent these substances from entering the brain. While primarily aimed at treating opioid use disorder, such vaccines could impact pain management by reducing abuse potential and overdose risk in patients with chronic pain [58,59]. These vaccines typically conjugate a small opioid molecule to a carrier protein, prompting the immune system to create antibodies that bind the drug in the bloodstream [58]. Vaccines against heroin, oxycodone, and fentanyl have shown efficacy in rodents [59,60,61]. For example, a heroin vaccine (hapten = morphine analog linked to CRM197 carrier) reduced heroin self-administration in rats and blunted heroin-induced analgesia and respiratory depression [61]. Similarly, mice immunized with a fentanyl vaccine failed to exhibit the normal antinociceptive response to fentanyl and were protected from lethal overdose [60,62]. Furthermore, heroin and fentanyl vaccines using a Qβ bacteriophage VLP carrier induced high-affinity antibodies in rodents that sequestered the drugs and blocked their analgesic and respiratory-depressant effects [59]. These findings demonstrate that an opioid vaccine can produce functionally significant antibody levels able to intercept opioid molecules in circulation before they reach opioid receptors in the CNS [59].

In terms of clinical evidence, a Phase 1 trial of an oxycodone vaccine in people with opioid use disorder reported that the vaccine was immunogenic, with higher doses yielding anti-oxycodone antibody titers that lasted several months [61,63]. Those antibodies selectively bound oxycodone and related opioids, and although the trial was not designed to test efficacy, there was suggestion of reduced drug liking in subjects who mounted strong antibody responses. In summary, opioid-targeted vaccines have demonstrated proof of concept in animals and initial safety in humans. Because these vaccines generate antibodies against opioids, they might help prevent misuse while also limiting the effectiveness of pain relief when opioids are medically necessary, for example, in cases of emergency surgery. Nonetheless, these studies represent the most advanced clinical experience with “anti-pain-drug” vaccines and lay groundwork for future therapeutic vaccine trials.

Cannabinoid vaccines are similarly being explored to treat cannabis misuse by inducing antibodies against delta-9-tetrahydrocannabinol (THC) or synthetic cannabinoids. In one study, a broad-spectrum vaccine was formulated to raise antibodies cross-reactive with multiple synthetic cannabinoid analogues [64]. Immunized mice developed antibodies that bound diverse cannabinoids, reducing their behavioral effects. Although these vaccines would neutralize exogenous cannabinoids rather than alleviate pain directly, they could be relevant to pain patients by preventing psychoactive cannabinoid abuse or by serving as adjuncts in pain treatment plans that aim to avoid cannabinoids. An alternative application is using vaccines to modulate endogenous opioid/cannabinoid pathways. For example, one could envision a vaccine that induces antibodies against β-endorphin or anandamide (endocannabinoid) to study or influence their role in chronic pain. Overall, vaccines targeting the opioid and cannabinoid systems represent a unique category, as they neutralize pain-relieving drugs rather than pain-causing molecules. Their primary role lies in addiction medicine and public health, but they intersect with pain management by potentially enabling safer use of analgesic agents. Future clinical deployment will need to carefully balance the benefits of reducing drug abuse with the ethical obligation to manage pain in vaccinated individuals.

## 3. Vaccine Platforms and Adjuvants

As highlighted above, developing effective pain vaccines requires innovative platforms to create a robust response against self-molecules or small drug haptens without compromising the physiologic role of the molecules or haptens. To this end, several vaccine platform technologies have been employed or proposed.

A unique challenge in pain vaccine development is that many leading targets—such as ion channels (e.g., Nav1.7, TRPV1) and other multi-pass membrane proteins—are difficult to present to the immune system in their native conformation. These proteins are embedded in lipid bilayers and often have small extracellular domains, making them challenging to express, purify, and stabilize in immunogenic form [65]. Incorrect folding or lack of native membrane context can lead to antibodies that bind the denatured protein but fail to recognize the functional target in vivo [66]. Likewise, soluble neuropeptides (e.g., CGRP, substance P) and cytokines (e.g., IL-6) are small, flexible, and poorly immunogenic, requiring multivalent display or carrier conjugation to break tolerance [67]. These physicochemical constraints strongly influence platform choice, adjuvant selection, and the likelihood of success, and must be considered alongside the underlying pain mechanism being targeted.

### 3.1. Peptide/Protein Conjugate Vaccines

Many pain-related targets (neuropeptides, small drug molecules) are poorly immunogenic on their own [68]. Conjugation to a large carrier protein can render them visible to the immune system. Common carriers include Keyhole Limpet Hemocyanin (KLH), diphtheria toxoid, or tetanus toxoid, which induce T-cell dependent immune responses [69]. For example, opioid vaccine studies used morphine analogues conjugated to KLH with an adjuvant to elicit anti-drug antibodies [70]. These conjugate vaccines are typically formulated with strong adjuvants (e.g., alum, monophosphoryl lipid A) to drive high-titer IgG responses [71].

### 3.2. Virus-like Particle (VLPs) Vaccines

VLPs are highly immunogenic nanoparticles (often ~25–100 nm) that present repetitive antigen arrays, effectively stimulating B cells [72]. This platform has shown success in breaking immune tolerance to self-molecules, a key challenge in the development of an anti-pain vaccine. In the NGF vaccine described earlier, researchers used a plant virus-like particle (derived from cucumber mosaic virus) as a carrier and chemically attached NGF to its surface [23]. The dense, multivalent display of NGF on the VLP helped elicit antibodies despite NGF being a self-protein. VLPs from bacteriophages (Qβ, AP205) have likewise been used to create opioid and CGRP vaccines (e.g., fentanyl hapten conjugated to Qβ VLP) [59]. VLP vaccines often induce stronger and longer-lasting antibody responses than soluble protein conjugates, and they typically do so without the need for additional adjuvant, as the VLP itself is able to activate pattern recognition receptors [73]. This platform’s versatility and potency make it a leading choice in newer anti-pain vaccine efforts [74].

### 3.3. DNA and mRNA Vaccines

Nucleic-acid vaccine technology offers a flexible way to induce immunity by having the host’s cells produce the antigen. Most recently, this technology was applied to the creation of the COVID-19 vaccine [75]. For pain targets, a DNA or mRNA vaccine could encode an immunogenic fragment of a receptor or secreted factor. For instance, an mRNA vaccine might encode a segment of Nav1.7 or TRPV1 engineered for secretion and fusion to an immune-stimulatory domain. The host would transiently produce the antigen and mount an immune response. While this approach has not yet been reported for pain applications, it could accelerate development because antigens can be designed and optimized in silico. One challenge is ensuring the antigen is presented in a way that breaks self-tolerance. Thus, the encoded antigen might include foreign T-helper epitopes or be encoded as a chimera with a highly immunogenic protein. mRNA and DNA vaccines also allow multiple pain targets to be combined in one vaccine (polyvalent constructs) that could be personalized based on a patient’s condition [64]. The safety profile of such vaccines in chronic use remains to be determined, but their adaptability is promising for future pain vaccine design [76].

### 3.4. Viral Vector Vaccines

Another platform uses replication-deficient viral vectors (e.g., modified adenovirus, adeno-associated virus) to deliver genes encoding the antigen [77]. This is essentially a long-acting gene therapy approach to vaccination. For example, a viral vector could carry a gene for a single-chain antibody that binds a pain mediator, turning the patient’s own cells into “antibody factories”. While conceptually intriguing, this blurs the line between vaccination and gene therapy and raises additional safety/regulatory complexities. It has not been attempted clinically for pain, to our knowledge, but could be explored in refractory cases in the same way viral vectors are used experimentally for chronic pain gene therapy [78].

### 3.5. Adjuvants

While there are many choices for platforms, as highlighted above, the choice of adjuvant is also critical for the development of efficacious anti-pain vaccines. Since many targets are self-antigens, strong adjuvants (tools like receptor agonists, emulsions, etc.) are often required to provoke a sufficient immune response. However, overly reactogenic adjuvants could cause excessive inflammation or autoimmune reactions [79]. New classes of adjuvants, like saponin-based nanoparticles or cytokine adjuvants, are being optimized to enhance antibody titers while minimizing adverse effects [80]. Additionally, targeted delivery systems (e.g., liposomes, emulsions) can favor certain response types (for instance, inducing more IgG vs. IgA depending on route). The route of immunization may also influence effectiveness. For example, opioid vaccines have been administered both intramuscularly and intranasally, with intranasal delivery preferentially inducing mucosal IgA in the nasopharynx, where inhaled drugs such as nasal fentanyl are more likely to gain entry [81].

In summary, vaccine platform selection is pivotal in anti-pain vaccine development. Conjugate and VLP-based vaccines have shown the most progress so far, enabling high antibody yields against difficult targets like NGF and opioids. Emerging nucleic acid technologies may further broaden what antigens can be effectively targeted. The optimal platform may also differ by target. For example, a small chemical like morphine requires a conjugate carrier, whereas a large protein like TRPV1 might be best delivered via DNA or VLP. Future research comparing these approaches head-to-head in animal models to identify which yields the strongest analgesic efficacy with acceptable safety is necessary.

**Figure 2:**
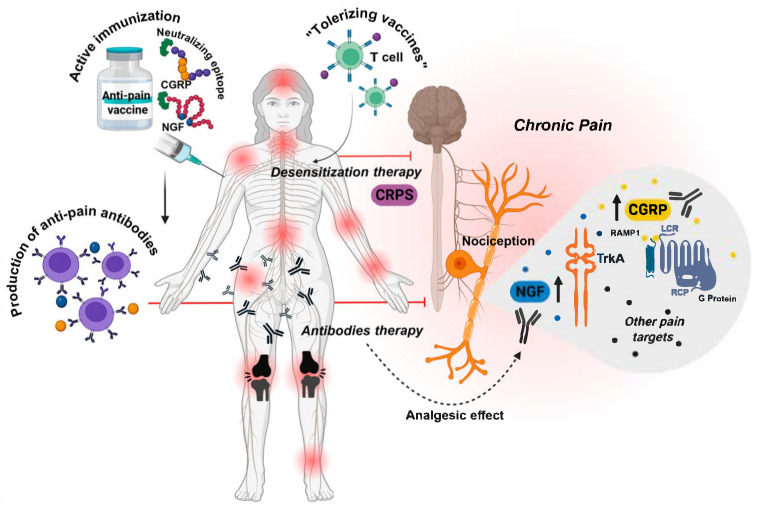
Immunological strategies for the treatment of chronic pain. Active immunization using anti-pain vaccines can stimulate the immune system to produce antibodies that target molecules involved in chronic pain, such as nerve growth factor (NGF) and calcitonin gene-related peptide (CGRP). These molecules play key roles in nociceptive signaling observed in conditions like osteoarthritis and migraine. By neutralizing these targets, vaccine-based therapies may provide longer-lasting analgesic effects compared to conventional medications. In addition to antibody-based approaches, a future therapeutic strategy may involve the induction of immune tolerance rather than a classical immune response. This could be achieved using tolerizing vaccines that modulate T cell activity. Such an approach may help control autoimmune-mediated pain conditions, including complex regional pain syndrome (CRPS), by dampening pathological immune responses.

A summary of the anti-pain vaccine targets and platforms can be seen in Figure 2.

## 4. Safety Considerations

Safety is a paramount concern in the development of vaccines for pain, given that many target endogenous molecules and are intended for use in non-life-threatening conditions, unlike traditional vaccines for infectious diseases. Several specific safety issues have been recognized within the literature.

### 4.1. Preclinical Observations

Generally, animal studies of pain vaccines have reported few acute safety concerns. In the NGF vaccine tested in mice, there were no signs of immune complex disease or neurological alterations [13]. In opioid-vaccinated rodents, behavior and organ histology have been normal aside from the intended pharmacologic blockade of the drug [82,83]. Autoimmune pathology has not been observed in short-term studies, even when targeting self-proteins like NGF or TRPV1 fragments [60]. That said, animal models may not capture subtle long-term effects; therefore, ongoing monitoring, such as testing for neural antibodies or off-target tissue binding, is an essential part of these preclinical programs. Encouragingly, many vaccines use platforms with prior human use (e.g., CRM197, alum adjuvant), providing some confidence in basic safety [61].

Overall, preclinical evidence strongly supports that vaccines can be generated to target pain pathways and that these immunizations can produce meaningful analgesic outcomes in animals without major side effects. Clinical evidence is still limited to migraine and addiction vaccine trials, and it also shows a good side effect profile [19,25,37]. The coming few years are likely to yield more data as first-in-human studies of anti-pain vaccines (for migraine, osteoarthritis, etc.) are initiated. These studies will be crucial to determine if the robust immunogenicity and efficacy seen in animals translate to humans dealing with chronic pain.

### 4.2. Autoimmune Responses

By design, vaccines against self-molecules break immune tolerance. This raises the risk of autoimmunity. Developers must ensure the induced antibodies (or T-cells) target the intended antigen specifically and do not cross-react with other proteins. For instance, NGF shares some functional overlap with other neurotrophins (brain-derived neurotrophic factor, neurotrophin-3), but sequence homology is low; so far, anti-NGF vaccines have not shown cross-reactivity to other neurotrophins in assays [84]. Nonetheless, continuous surveillance for autoimmune phenomena is needed. One theoretical concern is if a vaccine induces not just antibodies but also autoreactive T-cells that could attack tissues expressing the target. In the case of a TRPV1 vaccine, an autoimmune attack on TRPV1-positive neurons could cause sensory neuropathy [85]. Strategies to mitigate this include focusing the immune response on B-cell epitopes (antibody only) and avoiding T-cell epitopes common to human proteins. Modern epitope prediction and in vitro T-cell assays are used early in vaccine design to screen for problematic epitopes [85]. Additionally, some anti-pain vaccines use carriers such as VLPs or toxoids that present foreign T-helper epitopes. This reduces the reliance on self-peptides to elicit T-cell help and encourages the immune system to favor antibody production over cytotoxic T-cell responses.

### 4.3. Reversibility

Another safety aspect is that, unlike a drug, a vaccine’s effects cannot be quickly turned off. Antibodies may circulate for months or years, and if a patient experiences an adverse effect or if the target turns out to be essential for some physiological function, we cannot simply “unvaccinate”. For example, high titers of anti-NGF antibodies might theoretically interfere with nerve repair or development of fetal nervous tissue (hence, NGF vaccines would likely be contraindicated in pregnancy). Tanezumab trials revealed that excessive NGF blockade in adults can lead to accelerated arthropathy in damaged joints [86], possibly by masking pain and allowing overuse or by NGF’s trophic role in joint tissues. A vaccine could pose a similar risk if antibody titers are not carefully managed. To address this, protocols may use conservative dosing and allow antibody levels to wane if needed (since most antibody responses will gradually decline without boosters). In case of serious adverse events, techniques like plasmapheresis can remove antibodies from circulation, though this is an invasive measure. Therefore, safety trial designs should include stopping rules based on antibody titer thresholds and frequent monitoring of organ functions (e.g., joint imaging for NGF vaccines, liver enzymes for any off-target effects, etc.).

### 4.4. Off-Target Effects and Cross-Reactivity

Pain-related targets often have roles beyond pain. CGRP, for instance, helps regulate blood pressure and acts as a cardioprotective factor. Neutralizing it chronically could, in theory, increase hypertension or ischemic risk, although migraine mAb trials have so far not shown a major safety concern [37]. Nav1.7 is found in olfactory neurons, and individuals who lack functional Nav1.7 are unable to perceive smells [87]. Consequently, an aggressive Nav1.7 vaccine could cause anosmia or disturb other sodium channel functions if antibodies cross-react with Nav1.8 in the heart or central nervous system. When feasible, these possibilities could be evaluated in animal models and in early human trials through detailed sensory and functional testing. Still, a vaccine might produce higher or more sustained antibody levels, so continuous vigilance is required.

### 4.5. Immune Complex Deposition

If a vaccine induces very high antibody titers against a circulating antigen, there is a theoretical risk of forming antigen–antibody immune complexes that could deposit in tissues (like kidneys, blood vessels) and cause inflammation. Most pain targets are not present at high concentrations in the blood (NGF and CGRP circulate at low pg/mL levels), which minimizes this risk. In the NGF vaccine mouse study, even at peak antibody titers, there were no signs of immune complex disease [13]. Nonetheless, it remains a consideration, especially if patients were to receive multiple vaccines or already have autoimmune conditions. The choice of IgG subclass and maintaining a balance of antibody levels may help (IgG1 vs. IgG2 subclasses differ in complement activation potential, for example).

### 4.6. Local and Systemic Reactogenicity

Therapeutic vaccines often use potent adjuvants and may be given repeatedly, so the cumulative reactogenicity (injection site reactions, flu-like symptoms after each dose) is important. Patients with chronic pain might be particularly sensitive to additional flu-like side effects, which could transiently worsen pain or fatigue. Formulations must be optimized to minimize unnecessary inflammation while still inducing a strong, specific immune response. Emerging adjuvants like TLR7/8 agonists in nanoparticle form have shown an ability to enhance antibody responses to opioid haptens without dramatically increasing systemic cytokines [58]. Using such next-generation adjuvants could improve the tolerability profile of anti-pain vaccines.

### 4.7. Safety in Special Populations

If anti-pain vaccines are intended for chronic use, they may be given to populations including older adults (e.g., osteoarthritis patients) or those with comorbidities. In older patients, immunosenescence may impact vaccine effectiveness, potentially necessitating higher doses or the use of alternative adjuvants [88]. Patients with autoimmune disorders or immunodeficiencies also pose special challenges. An autoimmune patient might be at higher risk for a vaccine-triggered flare [89]. In contrast, an immunosuppressed patient may fail to generate an effective immune response, yet still be vulnerable to adverse effects from vaccine adjuvants [90].

In summary, developing and deploying vaccines for pain raises unique regulatory and ethical considerations. Unlike prophylactic vaccines for infectious diseases, therapeutic vaccines intended to treat a chronic pain condition have a different risk-benefit calculation since the recipients are patients, not healthy individuals, and the “benefit” (pain relief) is subjective. Although preclinical and early clinical data are encouragingly free of major red flags, safety oversight for anti-pain vaccines needs to be stringent. Risk mitigation strategies such as titrating dosage, comprehensive monitoring, and ensuring reversibility (to the extent possible) should be built into development programs. In addition, regulatory authorities will likely treat these therapeutic vaccines with heightened scrutiny on autoimmune potential. The goal should be to ensure that anti-pain vaccines are safe, especially given that alternative chronic pain treatments do exist.

Table 1 is a summary table comparing safety issues, platforms used, indications, and preclinical and clinical data across different anti-pain vaccine targets.

## 5. Regulatory and Ethical Issues

The development and implementation of anti-pain vaccines raise distinct regulatory and ethical challenges. Unlike prophylactic vaccines designed to prevent infectious diseases, these therapeutic interventions are intended to treat ongoing conditions. Consequently, risk-benefit assessments must account for the fact that recipients are patients actively seeking relief rather than healthy individuals. The inherently subjective nature of pain further complicates these evaluations [91].

Regulatory authorities such as the U.S. Food and Drug Administration and the European Medicines Agency are expected to evaluate anti-pain vaccines as rigorously as they do new analgesic medications and anti-cancer vaccines [92,93]. These vaccines occupy an intermediate space between conventional pharmaceuticals and biologics. To gain regulatory approval, developers must establish clearly defined efficacy endpoints, such as reductions in pain scores or improvements in physical function. Ideally, these should include objective measurements. A significant challenge lies in the high placebo response typical of pain-related trials, which necessitates tightly controlled and blinded study designs [94]. Although formal guidelines for therapeutic vaccines continue to evolve, precedents from areas such as cancer immunotherapy offer useful models [91]. Regulators are also likely to demand evidence linking immune responses with clinical outcomes. For example, showing that individuals with higher antibody titers experience greater pain relief could support efficacy claims [23]. Manufacturing considerations present additional complexity, as these vaccines often involve sophisticated biologics such as virus-like particles or conjugates. Ensuring lot-to-lot consistency, purity, and stability under current good manufacturing practices for biologics will be crucial [93]. Moreover, quality control may include functional assays to confirm immune activity, surpassing the requirements for standard small-molecule analgesics [95].

Ethical considerations are equally significant. Informed consent must be robust, given that anti-pain vaccines induce long-lasting immunological changes. Patients must be made fully aware that these vaccines may result in persistent antibody production and understand the implications. For instance, individuals vaccinated against opioid targets should be counseled that future opioid-based pain treatments might be less effective, potentially complicating care after surgery or injury [96]. Such individuals may need to carry documentation of their vaccination status. Respect for patient autonomy means no individual should feel pressured to receive an anti-pain vaccine. This issue is particularly acute in cases involving substances such as opioids. Offering the vaccine as a voluntary option is ethically distinct from requiring it as a prerequisite for receiving medication. For example, making an anti-pain vaccine a condition for continued opioid prescriptions would be ethically problematic, as it infringes on the patient’s right to make informed decisions [96,97]. Special care must be taken when considering vaccine use in vulnerable populations, such as those with substance use disorders or individuals in custodial settings.

In clinical practice, physicians must balance the ethical principles of beneficence and non-maleficence. The obligation to alleviate suffering must be weighed against the uncertainties surrounding novel anti-pain vaccines, including the potential for delayed or unforeseen adverse effects. Patients should be closely monitored, and clinicians must be prepared to respond to long-term complications such as autoimmune reactions [98]. Furthermore, if the vaccine fails to provide adequate analgesia, patients should not be left in worse circumstances. For example, someone who struggles with opioid dependence and receives an opioid-targeted vaccine may lose both the euphoric and analgesic effects of opioids. If the vaccine does not sufficiently manage the underlying pain, the patient may experience heightened distress. Therefore, anti-pain vaccines should be viewed as part of a broader, multimodal pain management strategy rather than as singular solutions. Within research settings, Data Safety Monitoring Boards play an essential role in maintaining ethical standards. They must be empowered to halt trials if participants are harmed or do not achieve meaningful relief [91].

Justice and equity concerns arise with respect to access and affordability. These vaccines may be expensive or require administration at specialized centers, raising questions about insurance coverage and availability. The typical justification for public funding of vaccines lies in their ability to prevent illness and reduce overall healthcare costs. In the context of chronic pain, the economic argument depends on reducing healthcare utilization and improving patient functionality. For instance, a migraine-targeted anti-pain vaccine that replaces the need for costly monthly monoclonal antibody infusions may offer long-term cost savings [23]. Decision-makers will need to weigh these considerations when determining reimbursement policies. Equitable access must also be addressed. Chronic pain affects people across all socio-economic levels, and pricing or distribution barriers could worsen existing disparities. However, if a single course of treatment yields multi-year benefits, these vaccines may ultimately improve access compared to long-term medication regimens. Policymakers may need to support subsidized access, particularly for patients such as elderly individuals with osteoarthritis who live on fixed incomes [96].

Designing ethical clinical trials for anti-pain vaccines introduces further challenges. Traditional Phase I trials often involve healthy volunteers, but exposing individuals without pain to vaccines that elicit auto-antibodies presents ethical concerns. As such, early-phase studies may recruit patients with the target condition, resulting in hybrid Phase I/IIa trial designs [91]. This approach necessitates careful ethical oversight to ensure participants are protected. If a participant’s pain worsens or adverse effects emerge, provisions must exist for rescue treatment and voluntary withdrawal. Control group design also warrants special consideration. Placebo arms are common in pain research, but in the context of vaccines, placebo recipients may receive no active treatment for extended periods. To address this, researchers may consider add-on study designs where all participants continue with their standard care while receiving either the vaccine or placebo as an adjunct therapy. This design helps ensure no participant is left without any therapeutic support [94]. Another challenge lies in maintaining study blinding. Immune responses such as local swelling or mild fever could inadvertently reveal group allocation. Strategies such as using active-control comparators may help preserve blinding integrity [94].

After regulatory approval, post-market surveillance will be critical. Authorities are likely to mandate long-term studies or registries to detect delayed or rare adverse events. For example, if an anti-nerve growth factor vaccine is approved, a multi-year registry could monitor recipients for neurological effects or other unintended consequences [95]. Healthcare providers must be trained in pharmacovigilance and encouraged to report adverse events promptly. Transparency with patients is essential throughout this process. They should be made aware that monitoring continues after approval and that treatment recommendations may evolve based on emerging data [95].

Product labeling for anti-pain vaccines will also need to address special populations and clinical scenarios. Labels may advise caution during pregnancy or suggest contraception for women of reproductive age around the time of administration, especially if the targeted molecule plays a role in fetal development [91]. For opioid-targeted vaccines, guidance may include alternative analgesics or recommendations for high-dose opioids or non-opioid agents such as ketamine. These considerations necessitate that clinicians and health systems recognize vaccine status in planning care. Potential solutions include adding alerts to electronic medical records or providing patients with identifiable documentation. Hospitals and emergency services may also need specific protocols for managing vaccinated individuals, including alternative anesthetic strategies. Regulatory agencies will be responsible for ensuring these scenarios are accounted for in clinical guidelines and that healthcare providers receive appropriate education and training.

In summary, the regulatory and ethical landscape for anti-pain vaccines is intricate but navigable. A patient-centered approach, combined with early engagement with regulatory bodies, can clarify key requirements such as immunological endpoints and acceptable risk profiles [92]. Ethical principles emphasizing informed consent, transparency, and protection of vulnerable populations must guide both clinical development and post-market implementation. As discussions around opioid-targeted vaccines and similar interventions continue, contributions from ethicists and public health experts will be essential in shaping policies that foster trust and ensure responsible integration of these therapies into pain management.

## 6. Future Directions

The field of anti-pain vaccines is in its infancy, and numerous avenues remain for research and innovation. As our understanding of pain mechanisms grows, additional molecular targets may be identified for vaccine development. Candidates could include inflammatory cytokines (e.g., TNF-α, IL-6) that contribute to pain. Indeed, a TNF-α vaccine has been tested in rheumatoid arthritis patients (a “TNF kinoid” showing reduced disease activity) [99]. Vaccination strategies targeting multiple pain mediators simultaneously may also be conceivable in the future, though this approach requires rigorous preclinical and clinical validation.

The heterogeneous nature of chronic pain suggests that a one-size-fits-all vaccine may not be ideal. In the future, we might personalize vaccine choice based on an individual’s pain biology. For example, patients with high NGF levels or specific polymorphisms in the NGF/TrkA pathway might respond especially well to an anti-NGF vaccine, whereas another subgroup with predominantly inflammatory pain could benefit more from a cytokine-targeted vaccine [100]. However, identifying reliable predictive biomarkers for vaccine responsiveness will necessitate extensive research and validation. In this context, artificial intelligence and machine learning applied to large datasets from pain cohorts could assist in patient stratification and matching to appropriate immunotherapies, though clinical evidence supporting such applications is currently limited [101].

Future vaccines could also benefit from advances in protein engineering and immunology. Stabilized epitope scaffolds, epitope-focused design, and novel carriers are areas of active development [102]. For instance, identifying a neutralizing epitope on NGF or CGRP could allow vaccines to induce a more focused antibody response, potentially increasing efficacy and reducing off-target immunity. Additionally, self-assembling nanoparticle platforms (beyond VLPs) are being created that can present antigens in highly ordered arrays with tunable spacing, which might optimize B-cell stimulation [103]. For small molecule targets (opioids, cannabinoids), chemists are synthesizing hapten designs that generate broader antibody coverage, so that a single vaccine can cover many analogues [64]. These approaches remain largely experimental and will require further validation in clinical settings.

Another emerging strategy is to use immunomodulators alongside vaccines to shape the immune response. For example, co-delivering an immune checkpoint inhibitor locally with a vaccine could amplify antibody production by transiently relieving B-cell inhibition. Previously, low-dose cyclophosphamide, which suppresses regulatory T-cells, was given with a peptide vaccine to increase its antibody titer response in a cancer setting [104]. Similar approaches might enhance pain vaccine responses. Moreover, given that chronic pain may require long-term management, strategies for periodic boosting will be important. One idea is a “prime-boost” approach where the initial priming is performed with one platform and boosting with another, which can sometimes strengthen and broaden immunity. Such heterologous prime-boost regimens are being tested in infectious disease vaccines like for HIV [105] or tuberculosis [106], and could be adapted for pain targets if initial immunity needs reinforcement.

While the focus thus far has been on antibody-mediated mechanisms, interesting evidence is emerging that T-cells also influence chronic pain [107]. Therefore, future pain vaccines might not only aim to produce antibodies but also modulate T-cell responses to pain-related antigens. For example, inducing regulatory T-cells (Tregs) that home to the nervous system and secrete anti-inflammatory cytokines could dampen pain. Tolerizing pain vaccines could also be explored. Tolerizing vaccines work by exposing the immune system to an antigen in a manner that promotes immune tolerance rather than triggering a typical immune response [108]. In autoimmune diseases, tolerogenic vaccines are being explored to turn off pathogenic T-cells [109]. Although this is a different paradigm (more akin to desensitization therapy than typical vaccination), the technologies overlap (e.g., using altered peptide ligands or specific adjuvants that drive tolerance).

Overall, the translational path for anti-pain vaccines is at an early and exploratory phase. Continued rigorous research, including well-designed clinical trials and biomarker studies, will be critical to establishing the safety, efficacy, and clinical applicability of these innovative approaches.

## 7. Public Perception and Education

Another consideration is how patients and the public will perceive pain vaccines. The term “vaccine” might cause confusion or undue concerns given its association with infectious disease prevention. Public education will be needed to clarify that these are optional therapeutic interventions for those suffering chronic pain, not something that will be mandated or given to everyone. Emphasizing the potential quality-of-life improvements and safety monitoring in place will be key to acceptance. Conversely, some patients may have overly high expectations (“a one-time vaccine to cure pain forever”); managing expectations realistically (it is not magic—boosters may be needed, etc.) will be part of physician counseling. Pain is a subjective and multifaceted experience, so any new treatment—especially one involving the immune system—should be introduced with empathy and clear communication.

## 8. Conclusions

In conclusion, the next decade is likely to bring significant developments in the anti-pain vaccine arena. We will see refinement of targets, smarter vaccine designs, and initial real-world data from pioneering trials like the migraine CGRP vaccine. If those are successful, it could open the floodgates for anti-pain vaccines targeting other conditions (arthritis, back pain, neuropathies). In the future, pain treatment may shift toward a more biologically targeted approach. Rather than relying on broadly acting analgesics, therapies could focus on the specific molecules responsible for an individual’s pain. This is aligned with the broader trend in medicine toward precision and personalization. It is a hopeful prospect for millions of patients who live with chronic pain—a field historically marked by trial-and-error treatments could finally have therapies that are not only effective and long-acting but also fundamentally alter the course of pain pathology for the better. Continued interdisciplinary collaboration between pain researchers, immunologists, and vaccine technologists will be essential to realize this promise in a responsible and impactful way.

## Figures and Tables

**Table 1:** Comparison of preclinical data, clinical data, safety issues, platforms used, and indications across different anti-pain vaccine targets.

Target	Preclinical Data	Clinical Data	Safety Issues	Platforms Used	Indications
NGF	VLP-NGF vaccine in mice showed high anti-NGF antibody titers, reversed pain behaviors in osteoarthritis model, no overt toxicity [23].	Phase I trial completed; good safety and tolerability; long-term efficacy still unknown [25].	Potential joint degeneration risk; long-term NGF blockade may affect repair mechanisms [17,86].	Virus-like particles (VLP) using cucumber mosaic virus [23].	Osteoarthritis, chronic low-back pain [17,23].
Substance P	Patent-based canine study with dual NGF+SP vaccine; strong antibody response and improved mobility; no peer-reviewed rodent data [32].	No human data; dual NGF+SP vaccine only tested in dogs (OA) [32].	Dual-targeting may increase complexity; off-target modulation of SPâ€™s anti-nociceptive roles is a concern [33].	Recombinant fusion protein vaccine combining SP and NGF for dogs [32].	Inflammatory/neuropathic pain (e.g., CRPS, fibromyalgia) [26,32].
CGRP	UB-313 vaccine generated high-affinity antibodies in rodents and primates; reduced pain behavior in trigeminal pain models [39].	UB-313 Phase 1 trial started 2023; interim data shows tolerability and antibody generation; efficacy pending [38].	Possible cardiovascular effects with long-term CGRP suppression; needs monitoring [40].	VLP platform UB-313, derived from bacteriophage VLPs [39].	Migraine (primary); potential for cluster headaches [38].
TRPV1	No direct TRPV1 vaccine tested; nanobody approaches modulate TRPV1; capsaicin-BSA vaccine raised antibodies in guinea pigs [51].	No clinical vaccine studies; hyperthermia remains a major safety barrier with TRPV1 targeting [46,47].	Hyperthermia and sensory disruption due to TRPV1â€™s thermoregulation role [46,47].	No vaccine yet; early-stage nanobody/immunogen work; capsaicin-BSA model [51].	Neuropathic and inflammatory pain syndromes [44].
Nav1.7	Nanobodies and mAbs reduced pain in rodent models; no vaccine yet; theoretical models propose peptide/domain-specific immunization [53,56].	No human data; theoretical risks include cross-reactivity and autoimmunity; no vaccine candidates yet [53,77].	Autoimmunity and anosmia due to Nav1.7’s expression in sensory/olfactory neurons [77].	Monoclonal/nanobody proof-of-concept; theoretical peptide/domain vaccines [53,56].	Congenital or acquired pain disorders; chronic inflammatory pain [53].
Opioids	Heroin/fentanyl/oxycodone vaccines showed protection in rodents; high antibody titers blocked analgesic and respiratory effects [59,62].	Phase 1 oxycodone vaccine showed immunogenicity in OUD patients; reduced drug liking in high responders [63].	Risk of impeding emergency analgesia; off-target immune complex disease not observed in short term [60,61].	Hapten-protein conjugates with KLH or VLPs (e.g., QÎ^2^); alum/MPL adjuvants [59].	Opioid use disorder; reducing abuse and overdose [58,59].
Cannabinoids	Synthetic cannabinoid vaccines in mice raised antibodies against various analogues; reduced behavioral effects; no pain data [64].	No clinical studies; potential for public health use in cannabis misuse rather than direct analgesia [64].	May interfere with therapeutic use of cannabinoids in legitimate medical contexts [64].	Synthetic cannabinoids conjugated to protein carriers; adjuvanted conjugate vaccines [64].	Cannabis misuse; public health and addiction contexts [64].

## Abbreviations

The following abbreviations are used in this manuscript:

BSA	Bovine Serum Albumin
CGRP	Calcitonin Gene-Related Peptide
CNS	Central Nervous System
COVID-19	Coronavirus Disease 2019
CRPS	Complex Regional Pain Syndrome
CRM197	Cross-Reactive Material 197
DNA	Deoxyribonucleic Acid
HIV	Human Immunodeficiency Virus
IASP	International Association for the Study of Pain
IgG	Immunoglobulin G
IL-6	Interleukin-6
KLH	Keyhole Limpet Hemocyanin
mAbs	Monoclonal Antibodies
mRNA	Messenger Ribonucleic Acid
Nav1.7	Voltage-Gated Sodium Channel Subtype 1.7
NGF	Nerve Growth Factor
NK1	Neurokinin-1 (Receptor)
NSAIDs	Nonsteroidal Anti-Inflammatory Drugs
PRRs	Pattern Recognition Receptors
Qβ	Qubevirus durum (a bacteriophage used as a VLP platform)
SCN9A	Sodium Voltage-Gated Channel Alpha Subunit 9
SP	Substance P
THC	Delta-9-Tetrahydrocannabinol
TLR	Toll-Like Receptor
TNF-α	Tumor Necrosis Factor-alpha
TRPV1	Transient Receptor Potential Vanilloid 1
Tregs	Regulatory T Cells
TrkA	Tropomyosin Receptor Kinase A
TTX	Tetrodotoxin
VLP	Virus-Like Particle
